# Chiral photochemistry of achiral molecules

**DOI:** 10.1038/s41467-022-29662-1

**Published:** 2022-04-19

**Authors:** Umberto Raucci, Hayley Weir, Christoph Bannwarth, David M. Sanchez, Todd J. Martínez

**Affiliations:** 1 Department of Chemistry and The PULSE Institute, Stanford University, Stanford, CA 94305 USA; 2 SLAC National Accelerator Laboratory, 2575 Sand Hill Road, Menlo Park, CA 94025 USA; 3 Present Address: Italian Institute of Technology, Genova, GE Italy; 4 Present Address: Institute of Physical Chemistry, RWTH Aachen University, Aachen, Germany; 5 Present Address: Design Physics Division, Lawrence Livermore National Laboratory, Livermore, CA USA

**Keywords:** Excited states, Molecular dynamics, Quantum chemistry

## Abstract

Chirality is a molecular property governed by the topography of the potential energy surface (PES). Thermally achiral molecules interconvert rapidly when the interconversion barrier between the two enantiomers is comparable to or lower than the thermal energy, in contrast to thermally stable chiral configurations. In principle, a change in the PES topography on the excited electronic state may diminish interconversion, leading to *electronically prochiral* molecules that can be converted from achiral to chiral by electronic excitation. Here we report that this is the case for two prototypical examples – cis-stilbene and cis-stiff stilbene. Both systems exhibit unidirectional photoisomerization for each enantiomer as a result of their *electronic prochirality*. We simulate an experiment to demonstrate this effect in cis-stilbene based on its interaction with circularly polarized light. Our results highlight the drastic change in chiral behavior upon electronic excitation, opening up the possibility for asymmetric photochemistry from an effectively nonchiral starting point.

## Abstract (web summary)

The authors report non-adiabatic first principles molecular dynamics to show how an achiral molecule can be converted to a chiral one upon photoexcitation. These results demonstrate the possibility of asymmetric photochemistry starting from achiral reactants.

## Introduction

**Fig. 1:**
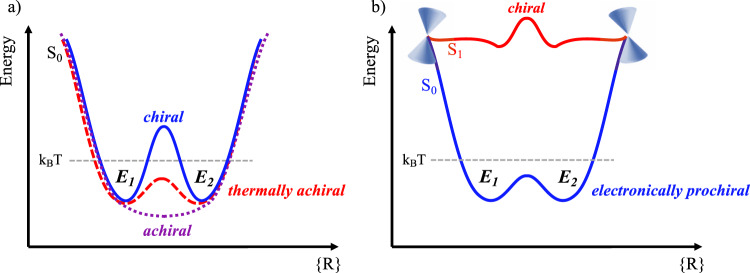
Schematic potential energy surfaces (PESs) for chiral and achiral molecules. **a** A barrier separates two minima corresponding to the two enantiomers (*E*_*1*_ and *E*_*2*_): if the interconversion barrier is much higher than the thermal energy (k_B_T), the molecule is chiral, otherwise rapid interconversion between the two enantiomeric configurations takes place making them inseparable (thermally achiral molecules). **b** PESs for electronically prochiral molecules which can be converted from achiral to chiral by electronic excitation. The example here shows a case where the molecule is thermally achiral on the ground state, but a similar scenario is also possible where the molecule is truly achiral on the ground state.

Chirality is a symmetry property widespread in nature, and examples include the molecules of life (e.g., proteins, nucleic acids). A molecule is chiral when it cannot be superimposed on its mirror image (enantiomer), and chirality can occur in asymmetric or dissymmetric systems (i.e., molecules that do not have any symmetry elements or do not have rotation-reflection axes). The effective (i.e., observable) chirality of a given molecule is governed by the topography of its potential energy surface (PES), specifically the height of the barrier separating the minima of the two enantiomers (the PES is symmetric with respect to an interconversion coordinate, Fig. 1). If the interconversion barrier is much higher than the thermal energy (k_B_T), the two enantiomers can be isolated and easily characterized (i.e., thermally stable chiral molecules, Fig. 1a). Otherwise, rapid interconversion between the two enantiomeric configurations takes place making them inseparable (i.e., thermally achiral molecules, Fig. 1a).

By tuning the PES topography it is possible to influence and control the inherent chiral behavior of the molecule. One such way is by promoting the molecule to an electronically excited state where the new electronic configuration can induce an increase/decrease of the interconversion barrier resulting in a shift of the enantiomeric ratio. Furthermore, the electronic excitation can introduce other accessible photoreactions (e.g., photoisomerization) which quench enantiomeric interconversion (Fig. 1b). In this case, the molecule can be considered electronically prochiral: it can be converted from achiral to chiral by electronic excitation. Electronic prochirality is a special case of the non-equilibration of excited state rotamers (NEER) principle where the two rotamers are effective enantiomers^1–4^. Applied mostly to photocyclization reactions (e.g., precalciferol, triene), the NEER hypothesis states that ring closure through a conical intersection occurs more rapidly than conformational changes due to an enhancement of the rotational barrier in the excited state.

Herein, we examine the concept of electronic prochirality via the relationship between enantiomer interconversion and *cis-trans* photoisomerization around a carbon–carbon double bond. In recent decades, this has been exploited to generate a local asymmetry in the PES resulting in unidirectional motion^5–13^. Indeed, the preferential selectivity of a particular direction of motion is the result of a nonequilibrium process in which the local topography of the PES is either sloped or characterized by a substantially lower energy barrier in that direction when compared to other possible pathways. The presence of chiral elements represents the simplest way to break the symmetry of the PES on the atomistic scale, ensuring that one rotational direction, e.g., clockwise (CW) or anticlockwise (ACW), is energetically preferred over the other.

Inspired by this principle, several generations of artificial light-driven molecular motors have been reported^5–13^. In these synthetic nanomachines, the introduction of asymmetric elements (e.g. stereogenic center or chirality axis) results in unidirectional rotation (CW or ACW) around a carbon–carbon double bond. Feringa and co-workers achieved full 360° unidirectional rotation by designing sterically overcrowded alkenes^6,8,9^. Here, a carbon–carbon double bond connects the stator and the rotor, and axial chirality is enforced by steric hindrance between the two halves. Bulky substituents in strategic positions around the rotational axis induce large twisting of the double bond, directing the olefinic bond rotation during the light-induced isomerization^6,9^. Marchand and co-workers reported that without axial chirality a single stereocenter in the allylic position of the isomerizing double bond is enough to ensure significant directionality in the photoisomerization^10^. Recently, Wang et al. exploited the axial chirality of cyclohexenylidene group to achieve unidirectional motion in a protonated Schiff-base^11^.

Here, we extend these findings to simple, highly symmetric molecules that are *thermally* achiral in the electronic ground state, where rapid interconversion between the two enantiomeric configurations takes place. Our simulations suggest that for electronically prochiral molecules, chirality can be enhanced or “stiffened” when the molecule is promoted to the excited state despite *thermal* achirality in the ground state. We demonstrate this effect on the *cis-trans* photoisomerization of 1,2-diphenylethylene (stilbene) and 1–1’-bis-indanylidene (stiff-stilbene).

## Results

**Fig. 2:**
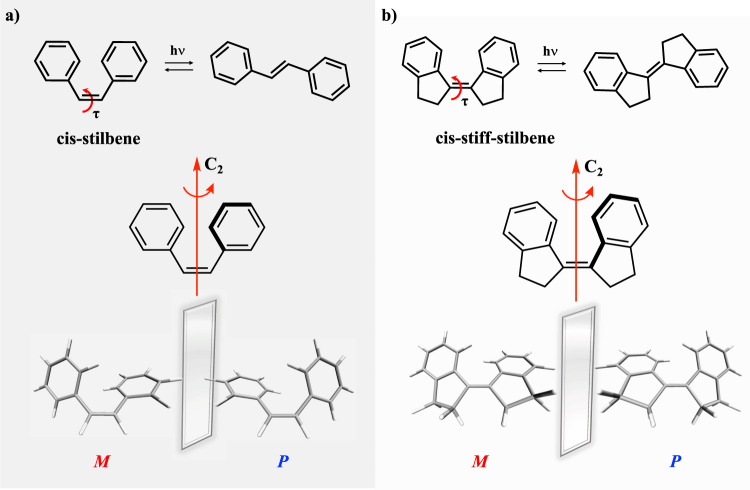
Schematic photochemistry and depiction of *P* and *M* enantiomers. *Cis*-stilbene is shown on the left (**a**) and *cis*-stiff-stilbene is shown on the right (**b**). The C_2_ rotational axis is highlighted in red along with the twist angle (*τ*) around the carbon–carbon double bond (defined as the average of the two dihedral angles involving the central C=C double bond).

Stilbene and stiff-stilbene represent prototypical examples of carbon–carbon double bond photoisomerization (Fig. 2). The steric hindrance between the phenyl groups prevents *cis*-stilbene and *cis-*stiff-stilbene from being completely planar in the ground electronic state (S_0_), resulting in a reduction in symmetry from C_2v_ to C_2_, with the C_2_ rotational axis perpendicular to the ethylene bond. Two helically twisted enantiomers arise for both *cis*-stilbene and *cis*-stiff-stilbene showing right-hand and left-hand helicity (*P* and *M,* respectively, Fig. 2).

Although the photoisomerization of stilbene and stiff-stilbene has been the topic of numerous theoretical and experimental studies over the past century^14–19^, to our knowledge the *cis-trans* photoisomerization dynamics have never been explored from the perspective of its rotational directionality. Here, we explore the rotational directionality in the non-adiabatic dynamics of the *P* and *M* enantiomers of *cis*-stilbene and *cis*-stiff-stilbene in the gas phase using a graphical processing unit (GPU)-accelerated multiconfigurational electronic structure theory in TeraChem^20–22^ coupled to ab initio multiple spawning (AIMS)^23–25^.

**Fig. 3:**
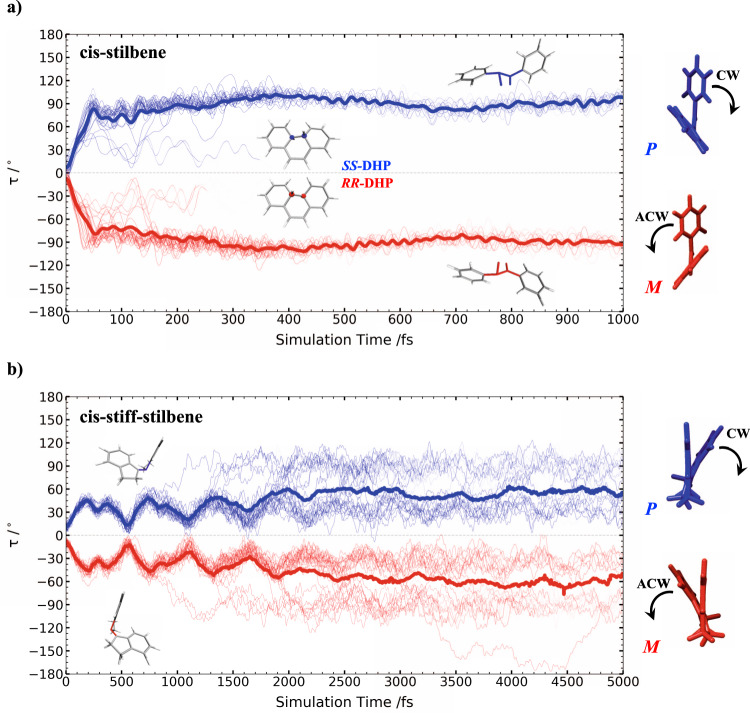
Time evolution of the twist angle (τ) on the S_1_ state for *P* (blue) and *M* (red) enantiomers. *Cis*-stilbene is shown in the upper panel (**a**) and *cis*-stiff-stilbene is in the lower panel (**b**). The bold line represents the weighted average of the total population over all the trajectory basis function (TBFs) on S_1_. Each line represents a TBF on S_1_, where the thickness of each line is proportional to its contribution to the weighted average.

To simulate the unidirectional photoisomerization in both *cis*-stilbene and *cis*-stiff-stilbene, AIMS trajectories for both *P* and *M* enantiomers are initiated from thirty initial conditions (positions and momenta) sampled from a 0 K harmonic Wigner distribution around their respective ground state minimum. Figure 3 shows the time evolution of the central ethylenic twist angle (*τ* in Fig. 2) around the photoisomerizing carbon–carbon bond for the two enantiomers (*P* and *M*) of *cis*-stilbene and *cis*-stiff-stilbene on the first singlet excited state PES, S_1_. We observe that the photoisomerization dynamics for each isomer are unidirectional: the evolution of the *P* enantiomer shows an increasing value of the *τ* angle until the *P* S_1_/S_0_ conical intersection (CI) region is reached at 90° (CW rotation), whereas the excitation of the *M* isomer leads to ACW rotation of *τ* to −90° at the *M* S_1_/S_0_ CI.

In addition to the photoisomerization around τ, *cis*-stilbene may also relax back to the ground state via photocyclization leading to 4a,4b-dihydrophenanthrene (DHP). The *cis-trans* isomerization and ring closure to DHP are orthogonal pathways accessed through distinct conical intersections^17,19,26–28^. As a consequence of the unidirectional dynamics, an enantioselective photocyclization is observed. Indeed, the CW rotation in (*P)*-*cis*-stilbene leads to (4a*S*,4b*S*)-4a,4b-dihydrophenanthrene (*S*,*S*-DHP), while (4aR,4bR)-4a,4b-dihydrophenanthrene (*R*,*R*-DHP) is formed from the ACW motion in (*M)*-cis-stilbene.

No *P-M* helical inversion is observed on S_1_ for both *cis*-stilbene and *cis*-stiff-stilbene indicating the occurrence of excited state axial chirality that leads to unidirectional photoisomerization. Hence, in spite of being formally achiral at thermal equilibrium on S_0_ due to the fast helical inversion process (inversion barriers are 1.6 and 4.9 kcal/mol for stilbene and stiff-stilbene, respectively, and transition state structures are shown in Supplementary Fig. 1), *cis*-stilbene and *cis*-stiff-stilbene behave as chiral molecules during their short life on S_1_. Indeed, the helical inversion barrier increases on S_1_ to 19 and 13 kcal/mol for stilbene and stiff-stilbene, respectively. As can be inferred from Supplementary Fig. 1, the helical inversion in *cis*-stilbene proceeds via phenyl rotation on the ground state, whereas the transition state is planar on the excited state, reached by ethylenic torsion. This behavior can be easily rationalized by inspecting the frontier molecular orbitals involved in the electronic excitation (Supplementary Fig. 2) as routinely done in organic photochemistry^29,30^. Indeed, the S_0_ → S_1_ transition corresponds to a HOMO → LUMO π-π* one-electron excitation characterized by a transfer of electron density from the central ethylenic bond to the π orbitals of the adjacent carbon pairs. The new electronic arrangement reached on S_1_ lowers the barrier for the rotation around the central carbon–carbon double bond and hinders the rotation of the phenyl groups necessary for the *P-M* inversion. The resulting unidirectional photoisomerization is thus observed as a result of a nonequilibrium process in the excited state, in which the local topography of the PES is sloped in the direction of the *cis-trans* isomerization compared to the helical inversion pathway (Fig. 1 and Supplementary Fig. 3).

Nevertheless, in order to achieve photo-induced *net* unidirectional motion, the preferential excitation of a specific enantiomer is required. Photoexcitation with non-polarized or linearly polarized light is unsuitable for this purpose because it would lead to a racemate in the excited state. In contrast, the differential absorption of circularly polarized light (CPL) results in bands with opposite signs in the electronic circular dichroism (ECD) spectrum for both enantiomers, meaning that an excess of one enantiomer is excited over the other. In Supplementary Fig. 4, we present the simulated relative absorption spectra for right circularly polarized light (r-CPL) of the (*M*)- and (*P*)-conformers of *cis*-stilbene. We use the quantum mechanically determined optical anisotropy Kuhn factor, *g*, (ratio of the dipole strength and the rotatory strength)^31^ for the S_0_ → S_1_ excitation to evaluate the enantiomeric excess of excited chiral species following r-CPL absorption (a more detailed discussion is reported in the SI). In the optical window 230–350 nm, the *M*-enantiomer preferentially absorbs the r-CPL, while the l-CPL is preferentially absorbed by the *P* conformer. As a consequence, the excitation of the ground state sample of *cis*-stilbene with r-CPL will preferentially excite the left-handed helical twisted *M*-enantiomer, whereas the *P* enantiomer can be preferentially excited with l-CPL.

**Fig. 4:**
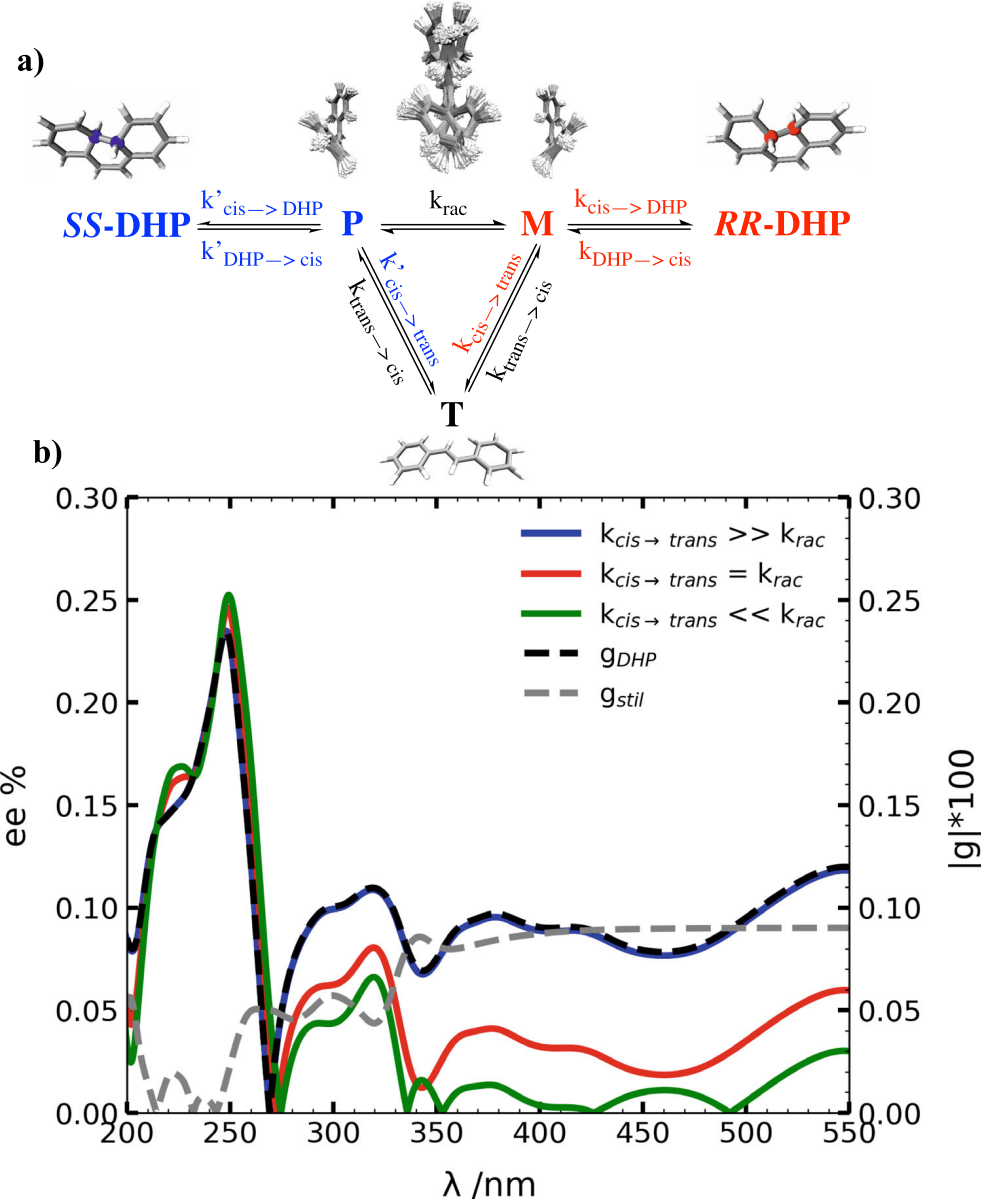
Asymmetric photochemistry in stilbene. **a** Kinetic model employed to describe the photochemistry of *cis*-stilbene. In agreement with the results of the AIMS dynamics, we are assuming that the *cis-trans* isomerization and the DHP cyclization require photoexcitation whereas the helical inversion process takes place only on S_0_. Thus, the excitation of the *M* and *P* enantiomers can lead to photocyclization to *R,R*-DHP or *SS*-DHP with kinetic constants *k*_*cis→DHP*_ and *k’*_*cis→DHP*_ or photoisomerization to the achiral *trans*-stilbene (T) with kinetic constants *k*_*cis→trans*_ and *k’*_*cis→trans*_, respectively. At the same wavelength, the DHP enantiomers can be excited back leading to the *M* and *P* enantiomers with kinetic constants *k*_*DHP→cis*_ and *k’*_*DHP→cis*_. *trans*-Stilbene can also be excited, producing both *P* and *M* *cis*-stilbene conformations (*k*_*trans→cis*_). *P* and *M* are considered to be in equilibrium in the ground state with a helical inversion constant *k*_*rac*_. **b** Enantiomeric excess reached at the photo-stationary state exciting a racemic mixture of the *P* and *M* enantiomers of *cis*-stilbene with r-CPL as a function of the excitation wavelength. Three limiting cases were considered according to Eq. (1). The wavelength-dependent *g* factors for stilbene (*g*_stil_) and DHP (*g*_DHP_) are also reported as dashed lines. In the optical window 250–350 nm, the *M*-enantiomer preferentially absorbs the r-CPL leading to trans-stilbene and *R*,*R*-DHP (Supplementary Fig. 4a). Th latter is also preferentially excited by the r-CPL retrieving *cis*-stilbene and resulting in an overall optical enrichment of *S,S*-DHP. On the other hand, in the optical window 200–270 nm (Supplementary Fig. 4b), the band in the DHP ECD spectrum changes sign and the r-CPL is preferentially absorbed by the *S*,*S*-DHP enantiomer (i.e., the anisotropy factor *g* changes sign). In this way, the optimum condition to maximize the *ee* is reached since one enantiomer is preferentially formed and the other destroyed by the CPL irradiation, resulting in an overall excess of *R,R*-DHP.

To supplement our findings for the unidirectional photoisomerization of *cis*-stilbene, we simulated a rather straightforward experiment based on the enantioselective photocyclization leading to *S*,*S*-DHP and *R*,*R*-DHP. Indeed, the excitation of *cis*-stilbene (existing as a racemic mixture of the *P* and *M* enantiomers at thermal equilibrium) with non-polarized UV light will lead to a racemic mixture of the chiral DHP photoproduct, whereas an excess of one enantiomer over the other is expected with the CPL excitation. Due to the fact that DHP can absorb CPL in the same optical window as *cis*-stilbene, the enantiomeric excess (*ee*) of DHP reached at the photo-stationary state can be predicted considering the kinetic model reported in Fig. 4a. In agreement with the results of the AIMS simulations, we assume that the *cis-trans* isomerization and the DHP cyclization require photoexcitation whereas the helical inversion process takes place only on S_0_. At the photo-stationary state, the enantiomeric excess of DHP can be expressed as follows (the complete derivation is discussed in the **SI**):

1 $$ee=\frac{|[S,SDHP]-[R,RDHP]|}{[S,SDHP]+[R,RDHP]}=\frac{|{k}_{cis\to trans}({g}_{{{{{{\mathrm{DHP}}}}}}}-{g}_{{{{{{\mathrm{stil}}}}}}}{g}_{{{{{{\mathrm{DHP}}}}}}})+2{k}_{rac}({g}_{{{{{{\mathrm{DHP}}}}}}}-{g}_{{{{{{\mathrm{stil}}}}}}})|}{{k}_{cis\to trans}(1-{g}_{{{{{{\mathrm{stil}}}}}}})+2{k}_{rac}(1-{g}_{{{{{{\mathrm{stil}}}}}}}{g}_{{{{{{\mathrm{DHP}}}}}}})}$$

where *g*_stil_ and *g*_DHP_ are the *g* factors of *cis*-stilbene and DHP, respectively (Supplementary Fig. 4), while *k*_*cis→trans*_ and *k*_*rac*_ represent the kinetic constants of the *cis-trans* photoisomerization reaction, and the ground state helical inversion process, respectively. The above expression demonstrates how the *ee* depends on the asymmetry of the electronic excitations (via the *g* factors), the *cis*-*trans* photoisomerization, and the ground state helical inversion processes.

For a given set of *g* factors, three limiting cases arise: the *cis-trans* photoisomerization is faster than the ground state helical inversion (*k*_*cis→trans*_ » *k*_*rac*_), S_0_ racemization is faster than the *cis-trans* isomerization (*k*_*rac*_ » *k*_*cis→trans*_) or these processes take place on the comparable time scale (*k*_*cis→trans*_ ~ *k*_*rac*_). The wavelength-dependent enantiomeric excess of DHP is reported for the three cases in Fig. 4b. According to our simulation, the maximum enantiomeric excess reached around 250 nm is ~0.23% when the photoisomerization is faster than the helical inversion process (which is the case for *cis*-stilbene due to its sub-picosecond *cis-trans* photoisomerization^14–19^), whereas it is ~0.25% in the other two limit cases. The predicted *ee* is comparable with what Butchardt and co-workers reported for the asymmetric synthesis of chiral helicenes with CPL^32–34^. In these early experiments, the different CPL absorption of the enantiomeric conformations of the diarylethylene substrate led to an optical yield of about 0.2%^33,34^. More recently, Feringa and co-workers were able to induce asymmetric photoisomerization in sterically overcrowded alkenes where they reached an *ee* of 0.07%^35–37^ by enantioselectively exciting a racemic mixture of thermally stable enantiomers (free enthalpy of racemization larger than 20 kcal/mol) with CPL leading to unidirectional photoisomerization.

Our kinetic model shows that the expected enantiomeric excess is strongly dependent on the *g* factors. Considering that the anisotropy factors are typically quite small (less than 0.01) a low *ee* should be expected^32^. Future improvements may include the excitation to states with larger *g* factors as well as shifting of the photo-stationary equilibrium, e.g., by selective removal of one of the enantiomeric photoproducts. Indeed, the optimal condition to maximize the *ee* is reached when one enantiomer is preferentially formed and the other destroyed by the same monochromatic CPL irradiation (corresponding to *g* factors with opposite sign, see Supplementary Fig. 6 for a generic A → B photoreaction). Since the anisotropy factors are wavelength-dependent, the aim is to find the optical window that maximizes the excitation asymmetry.

In conclusion, our non-adiabatic simulations of stilbene and stiff-stilbene suggest that electronic excitation can change the inherent chiral behavior of molecules. We coin the term electronically prochiral to describe molecules whose enantiomer interconversion can be hindered in the excited electronic state, opening up the possibility for asymmetric photochemistry from an effectively nonchiral starting point. Exploiting such asymmetric photochemistry and its connection with unidirectional motion will represent the next step toward designing new generations of responsive smart materials.

## Methods

### Computational details

The excited state non-adiabatic dynamics of *cis*-stilbene and *cis*-stiff-stilbene were investigated with ab initio multiple spawning^23–25^ interfaced with multiconfigurational electronic structure methods. GPU accelerated State-Averaged Complete Active Space Self-Consistent Field (SA-2-CASSCF(2,2)/6-31 G*) was employed to model the photochemistry of *cis*-stilbene. This level of theory has been shown to provide a reliable description of *cis*-stilbene photoisomerization^17,19^, agreeing well with experimentally determined lifetimes and branching ratios. For example, the computed branching ratio of 44:52:4 for the three photoproducts (*cis*-stilbene, *trans*-stilbene, and DHP)^14–19^ is in line with recent experiments using transient absorption spectroscopy with 318 nm excitation wavelength, which yielded 5% DHP^38^.

Modelling the excited state landscape of *cis*-stiff-stilbene has been shown to be a challenging task^14^. Here, we employ the α-scaled Floating Occupation Molecular Orbital Complete Active Space Configuration Interaction (α-FOMO-CASCI) electronic structure method^39–41^, which provides a balanced description of the twisting barriers on both the *cis* and *trans* side. The main idea behind α-FOMO-CASCI is to recover effects on the electronic state splitting arising from dynamic electron correlation that is mostly absent in the FOMO-CASCI wavefunction through an α scaling of the state-specific energy splitting while leaving the state-average energy untouched in the same way as α-CASSCF^41^. Adopting α(0.8)-FOMO(β = 0.2)-CAS(2,2)CI/6-31 G* (where α is the scaling factor, and β represents the FON temperature) with two electrons into two π orbitals, the computed ratio of the *cis* and *trans* barriers shows good agreement with experimental estimates. Critical points of the ground and excited-state potential energy surfaces are reported for *cis*-stiff-stilbene in Supplementary Fig. 7. The TeraChem electronic structure package^20–22^ was employed to perform all the electronic structure calculations.

### Initial conditions generation

Thirty initial conditions (ICs) for each enantiomer (*P* and *M*) of *cis*-stilbene and *cis*-stiff-stilbene were selected out of 500 geometries sampled from a 0 K harmonic Wigner distribution corresponding to geometry and frequencies computed at B3LYP/6-31 G* and B3LYP/6-31 G** for *cis*-stilbene and *cis*-stiff-stilbene, respectively. These 500 phase space points were used to simulate the electronic absorption spectra (Supplementary Fig. 8). The absorption spectra were generated by single-point energy calculations at SA-2-CASSCF(2,2)/6-31 G* and α(0.8)-FOMO(β = 0.2)-CAS(2,2)CI/6-31 G*, for *cis*-stilbene and *cis*-stiff-stilbene, respectively. The final spectra were obtained by broadening the S_0_ → S_1_ excitation energies with Gaussian functions (full-width half maximum of 0.2 eV). We randomly selected 30 different ICs from the 500 phase space points used to simulate the spectra. These ICs (positions and momenta) were placed on the S_1_ surface and propagated with AIMS.

### *P*-*M* thermal barriers

Transition states (TS) for the *P-M* helical inversion were evaluated by the Transition State finder in DL-FIND through the ChemShell/TeraChem interface. B3LYP-D3/def2-TZVP(-f)^42–44^ was employed. The TS involved in the helical inversion is reported in Supplementary Fig. 1.

### Simulation of circular dichroism spectra

The electronic absorption and ECD spectra (Supplementary Fig. 9) were computed by means of the simplified time-dependent density functional theory (sTD-DFT)^45,46^. We used 1000 structures from the aforementioned 0 K harmonic Wigner distribution. At each of these structures, we computed Kohn-Sham density functional theory single-point energies with BHLYP/def2-TZVP(-f)^44,47–49^ using the TeraChem program. The molecular orbitals and orbital energies were then used to compute all vertical excitations up to 10 eV with the sTD-DFT method as implemented in the sTDA program (v1.6) (https://github.com/grimme-lab/stda). All excitations were blue-shifted by 0.7 eV and convolved with Gaussians of 0.24 eV width at 1/e maximum to match the previously reported experimental absorption (in hexane)^50^. The ECD spectra and the relative r-CPL absorption spectra were obtained and shifted accordingly. The spectroscopic data for stiff-stilbene was determined in the same way. A blue shift of 0.35 eV was used to match the absorption spectrum from ref. ^50^.

## Supplementary information

Supplementary Information

## Data Availability

Data generated in this study have been deposited in Zenodo (https://zenodo.org) under the accession code 10.5281/zenodo.6430143.

## Code availability

Results described in this manuscript have been obtained with publicly available software packages such as TeraChem (http://www.petachem.com), MolPro (http://www.molpro.net), and sTDA (https://github.com/grimme-lab/stda).
