# Supplementary material for: Chiral photochemistry of achiral molecules
Source: Nat Commun. 2022 Apr 19;13:2091. doi: 10.1038/s41467-022-29662-1 (PMC9019051; doi:10.1038/s41467-022-29662-1)
Supplement: Supplementary file 1 — Supplementary Information [file 41467_2022_29662_MOESM1_ESM.pdf]

Supplementary Information

Chiral Photochemistry of Achiral Molecules

U. Raucci, et al.

Supplementary Discussion

Helical inversion: Two achiral transition states (TS) have been identified for the helical inversion process in cis-stilbene: a geometry with C_s symmetry characterized by the perpendicular arrangement of the two phenyl groups and a completely planar structure with C_{2v} symmetry. The C_s geometry is lower in energy on S_0, and an energy difference of 6.1 kcal/mol has been computed between the C_s and C_{2v} transition states at B3LYP-D3/def2-TZVP(-f) level of theory. On S_1 the planar C_{2v} structure is 8.4 kcal/mol more stable than the C_s one, according to TD-B3LYP-D3/def2-TZVP(-f). We also verified the S_1 relative energy between these two structures with a range-separated hybrid functional (TD-ωB97X-D3/def2-TZVP(-f)) obtaining a similar (but somewhat increased) ΔE of 10.2 kcal/mol. The increased stability of the planar geometry in the excited state can be easily rationalized by inspecting the frontier molecular orbitals involved in the electronic excitation (**Supplementary Figure 2**). Indeed, the $S_0 \rightarrow S_1$ transition corresponds to a HOMO $\rightarrow$ LUMO π-π^* one electron excitation characterized by a transfer of electron density from the central ethylenic bond to the π orbitals of the adjacent carbon pairs (**Supplementary Figure 2**). Thus, on S_1 the twisting of the phenyl group to helically invert becomes more energetically costly than twisting around the ethylenic bond through a planar intermediate.

Nudged elastic band (NEB) calculations from the Franck-Condon point to the planar transition state have been performed on S_1 to estimate the excited state helical inversion barrier (**Supplementary Figure 3** and **Supplementary Table 1**). We also performed NEB from the Franck-Condon point to the twisted S_1 minimum.

The NEB calculations clearly show that the S_1 PES is sloped in the direction of the cis-trans photoisomerization compared to the helical inversion pathway. Starting from the Franck-Condon point, the system evolves toward the planar transition state passing from the S_1 minimum.

A consistent helical inversion barrier of about 0.8 eV (~19 kcal/mol) has been computed at TD-DFT (TD-B3LYP-D3 and TD-ωB97X-D3), CASSCF and XMS-PT2 levels of theory.

In cis-stiff-stilbene the helical inversion goes through a planar transition state on both S_0 and S_1. The helical inversion barrier on S_1 is about 0.5 eV (13 kcal/mol at TD-B3LYP-D3 and 14 kcal/mol at α-FOMO-CASCI level of theory).

Simulation of Circular Dichroism spectra: The electronic absorption and circular dichroism (ECD) spectra were computed by means of the simplified time-dependent density functional theory (sTD-DFT).[1,2] We used 1000 structures from the aforementioned 0 K harmonic Wigner sampling based on the geometry and harmonic frequencies computed at B3LYP/6-31G* level of theory. At each of these structures, we carried out Kohn-Sham density functional theory single-point energy calculations at the BHLYP/def2-TZVP(-f)[3-6] level of theory with the TeraChem program. The molecular orbitals and orbital energies were then used to compute all vertical excitations up to 10 eV with the sTD-DFT method as implemented in the sTDA program (v1.6).[7] All excitations were blue-shifted by 0.7 eV and convolved with Gaussians of 0.24 eV width at 1/e maximum to match the experimental absorption[8] (in hexane). The same settings were used to simulate the ECD spectra and the relative right circularly polarized light (r-CPL) absorption. We work with the velocity form of the rotatory strengths, which is computed for the (*M*)-conformer of *cis*-stilbene.

The r-CPL absorption is given by:[9]

$$\varepsilon_{+}^{(M)}(\omega) \propto \sum_{0\rightarrow A} \rho_{0\rightarrow A}(\omega)\left(D_{0\rightarrow A}^{(M)} - 2R_{0\rightarrow A}^{(M)}\right) \tag{1}$$

Here, $D_{0\rightarrow A}$ is the quantum mechanical dipole strength and $R_{0\rightarrow A}$ is the rotatory strength for the excitation from the ground to an excited state *A*. $\rho_{0\rightarrow A}(\omega)$ is the transition density for this

transition at a given excitation energy ω. For a quantitative discussion, the dipole and rotatory strengths have to be brought into the same unit system, which is commonly done by bringing them to cgs units.

We plotted the r-CPL absorption, in relative units normalized to the half of the summed r-CPL and l-CPL absorption at 275 nm, i.e., relative to $\frac{2\varepsilon_+^{(M)}}{\varepsilon_+^{(M)} + \varepsilon_-^{(M)}} = 2\frac{\varepsilon_+^{(M)}}{\varepsilon_+^{(M)} + \varepsilon_+^{(P)}}$.

The dissymmetry factor (g) has been computed as:

$$\left|g^{(M)}\right| = \frac{\left|\varepsilon_+^{(M)} - \varepsilon_+^{(P)}\right|}{\varepsilon_+^{(M)} + \varepsilon_+^{(P)}} = \frac{\left|\Delta\varepsilon^{(M)}\right|}{\varepsilon} = 4\frac{\left|R_{0\to A}^{(M)}\right|}{D_{0\to A}} \quad (2)$$

Kinetic model: Kinetic modeling was employed to describe the photochemistry of cis-stilbene. To simplify the following notation, the kinetic constants are labeled in **Supplementary Figure 5** as k_n. k_1, k_2, k_3, k_4, k_5, k_6, k_7, and k_8 corresponding to the kinetic constants, $k'_{cis\to DHP}$, $k'_{DHP\to cis}$, $k_{cis\to DHP}$, $k_{DHP\to cis}$, $k'_{cis\to trans}$, $k_{trans\to cis}$, $k_{cis\to trans}$, *and* k_{rac} in **Figure 4a**, respectively. In agreement with the results of the AIMS dynamics, we are assuming that the cis-trans isomerization and the DHP cyclization require photoexcitation whereas the helical inversion process takes place only on S_0. Thus, in this simplified model, the excitation of the *P* and *M* enantiomers can lead to photocyclization to *S,S*-DHP or *RR*-DHP with kinetic constants k_1 and k_3 or photoisomerization to the planar achiral trans-stilbene (T) with kinetic constants k_5 and k_7, respectively. At the same wavelength, the DHP enantiomers can be excited back leading to the *P* and *M* enantiomers with kinetic constants k_2 and k_4. Trans-Stilbene can also be excited, producing both *P* and *M* cis-stilbene conformations (k_6). Furthermore, *P* and *M* are considered to be in equilibrium with a helical inversion kinetic constant k_8.

The following kinetic equations describe the time variation of the *P*, *M*, *SS*-DHP, *RR*-DHP and T concentration:

$$\frac{d[P]}{dt} = -k_1[P] + k_2[SS-DHP] - k_5[P] + k_6[T] - k_8[P] + k_8[M] \tag{3}$$

$$\frac{d[M]}{dt} = -k_3[M] + k_4[RR-DHP] - k_7[M] + k_6[T] - k_8[M] + k_8[P] \tag{4}$$

$$\frac{d[SS-DHP]}{dt} = -k_2[SS-DHP] + k_1[P] \tag{5}$$

$$\frac{d[RR-DHP]}{dt} = -k_4[RR-DHP] + k_3[M] \tag{6}$$

$$\frac{d[T]}{dt} = -2k_6[T] + k_5[P] + k_7[M] \tag{7}$$

At the photo-stationary state:

$$\frac{d[P]}{dt} = \frac{d[M]}{dt} = \frac{d[SS-DHP]}{dt} = \frac{d[RR-DHP]}{dt} = \frac{d[T]}{dt} = 0 \tag{8}$$

Solving equations (3)-(7) at the photo-stationary state leads to:

$$[SS-DHP] = \frac{[T]k_1k_6(k_7 + 2k_8)}{k_2(k_5k_7 + k_5k_8 + k_7k_8)} \tag{9}$$

$$[RR-DHP] = \frac{[T]k_3k_6(k_5 + 2k_8)}{k_4(k_5k_7 + k_5k_8 + k_7k_8)} \tag{10}$$

The DHP enantiomeric excess reached at the photo-stationary state can thus be expressed as:

$$ee_{DHP} = \frac{[SS-DHP] - [RR-DHP]}{[SS-DHP] + [RR-DHP]} = \frac{k_1k_4k_7 + 2k_1k_4k_8 - k_2k_3k_5 - 2k_2k_3k_8}{k_1k_4k_7 + 2k_1k_4k_8 + k_2k_3k_5 + 2k_2k_3k_8} \tag{11}$$

In the case of excitation with non-polarized UV-light k_1 is equal to k_3, k_2 to k_4 and k_5 to k_7; the reaction kinetics will be totally symmetric, and no enantiomeric excess is obtained.

The excitation with CPL light creates an asymmetry in the reaction kinetics according to the anisotropy factors of cis-stilbene (g_{stil}) and DHP (g_{DHP}). Indeed, k_1 can be related to k_3 by the g_{stil} factor, k_2 to k_4 by g_{DHP} and k_5 to k_7 again by g_{stil}. More in details, assuming that the quantum yield of the photochemical reactions is the same for both the *P*/*M* and *SS*-DHP/*RR*-DHP enantiomers, we can express the k_1/k_3, k_2/k_4, and k_5/k_7 ratios as follows:

$$\frac{k_1}{k_3} \propto \frac{\varepsilon_+^P}{\varepsilon_+^M} \propto \frac{1-g_{stil}}{1+g_{stil}} \tag{12}$$

$$\frac{k_2}{k_4} \propto \frac{\varepsilon_+^{SSDHP}}{\varepsilon_+^{RRDHP}} \propto \frac{1-g_{DHP}}{1+g_{DHP}} \tag{13}$$

$$\frac{k_5}{k_7} \propto \frac{\varepsilon_+^P}{\varepsilon_+^M} \propto \frac{1-g_{stil}}{1+g_{stil}} \tag{14}$$

where we use equation (2) to relate the $\varepsilon_P/\varepsilon_M$ and the g factor. Inserting relations (12)-(14) into equation (11) leads to:

$$ee_{DHP} = \frac{k_7(g_{DHP} - g_{stil}g_{DHP}) + 2k_8(g_{DHP} - g_{stil})}{k_7(1-g_{stil}) + 2k_8(1-g_{stil}g_{DHP})} \tag{15}$$

Electronic structure validation. We detail the validation of the electronic structure theory used for both *cis*-stilbene and *cis*-stiff-stilbene.

Cis-stilbene: State-Averaged Complete Active Space Self-Consistent Field (SA-CASSCF(N,M) where N and M are the number of electrons and orbitals, respectively) was employed to model the photochemistry of cis-stilbene.

We recently validated the ability of the chosen method (SA-2-CASSCF(2,2)/6-31G*) to describe cis-stilbene photochemistry by (a) comparison of critical point energies and geometries

(including ground and excited state minima and conical intersections) to MS-CASPT2/SA-3-CASSCF(2,2)/6-31G* calculations including dynamic correlation effects and (b) direct comparison to experimental data such as the absorption spectrum, photoproduct branching ratio, and excited state lifetime.[10] In agreement with previous work,[11] we find that the qualitative features of the PESs are in reasonable agreement between SA-2-CASSCF(2,2) and MS-CASPT2/SA-3-CASSCF(2,2). Furthermore, the excited state dynamics agree well with experimentally determined lifetimes and branching ratios. We computed a branching ratio of 52.0 ± 3.6% trans-stilbene, 44.5 ± 3.7% cis-stilbene, and 3.5 ± 1.4% DHP, which is in line with recent experiments using transient absorption spectroscopy with 318 nm excitation wavelength, which yielded 5% DHP.[12] The excited state lifetime of cis-stilbene computed from our dynamics simulations is 520 ± 40 fs in line with the 320 fs decay time obtained from a time-resolved multiphoton ionization experiment.[13] For the direct comparison of critical point energies and geometries at SA-2-CASSCF(2,2) and MS-CASPT2/SA-3-CASSCF(2,2) and for the comparison with experimental results please refer to Ref. 10 and its Supplementary Information.

Cis-stiff-stilbene: Modelling the excited state landscape of cis-stiff-stilbene has been shown to be a challenging task. The main issue regards the correct description of the excited state landscape. Transient absorption data in hexane suggest that a torsional barrier of 2.4 kcal/mol exists between the cis S_1 minimum and the 90° twisted geometry.[14] At the same time, a smaller barrier of about 1.8 kcal/mol is present on the trans side between the trans S_1 minimum and the twisted arrangement. Time Dependent Density Functional Theory calculations were performed by Improta and Santoro,[15] showing a barrierless twist for the trans isomer, while a small barrier (50-100 cm^{-1}) was computed for the cis-stiff twisting. Opposite results were obtained by Liu and Morukuma with 6SA-CAS(10,10)SCF: a small barrier was computed for the trans isomer while no barrier was

found on the cis side.[16] Quick and co-workers used extended multiconfigurational quasi-degenerate second order perturbation theory to predict a quite small barrier for the cis-stiff twisting and barrierless torsion for trans-stiff.[14] We adopted the α-scaled state averaged Floating Occupation Molecular Orbital Complete Active Space Configuration Interaction (α-FOMO-CASCI) method, which provides a balanced description of the twisting barriers on both the cis and trans side.

Adopting α(0.8)-FOMO(β=0.2)-CAS(2,2)CI/6-31G* (where α is the scaling factor, and β represents the FON temperature in atomic units) with two electrons in two π orbitals, we computed a S_1 twisting barrier of 5.3 kcal/mol for cis-stiff and a value of 3.4 kcal/mol on the trans side. Furthermore, the ratio of the cis and trans barriers (0.64) is in line with the experimental ratio of about 0.75,[14] and this is reflected in a faster photoisomerization dynamics for trans-stiff compared to cis-stiff (unpublished results). Critical points along the photoisomerization pathway are reported in **Supplementary Figure 7**.

Supplementary Tables

Supplementary Table 1. S_0 and S_1 helical inversion barrier (kcal/mol) computed for stilbene and stiff-stilbene. On S_0 the barrier is compute as the energy difference between the S_0 minimum and the transition state. On S_1, the barrier is computed from the NEB between the Franck-Condon point and the TS structure as the energy difference between the lowest energy image and the transition state.

	Stilbene	Stiff-Stilbene
**S_0**		
B3LYP	1.60	4.93
**S_1**		
B3LYP	18.96	13.23
ωB97x-D3	18.66	14.26
SA-2CASSCF(2,2)†	19.42	14.19
3-XMS(2,2)-PT2	20.91	15.24

†αFOMO-CAS(2,2)CI for stiff-stilbene

Supplementary Figures

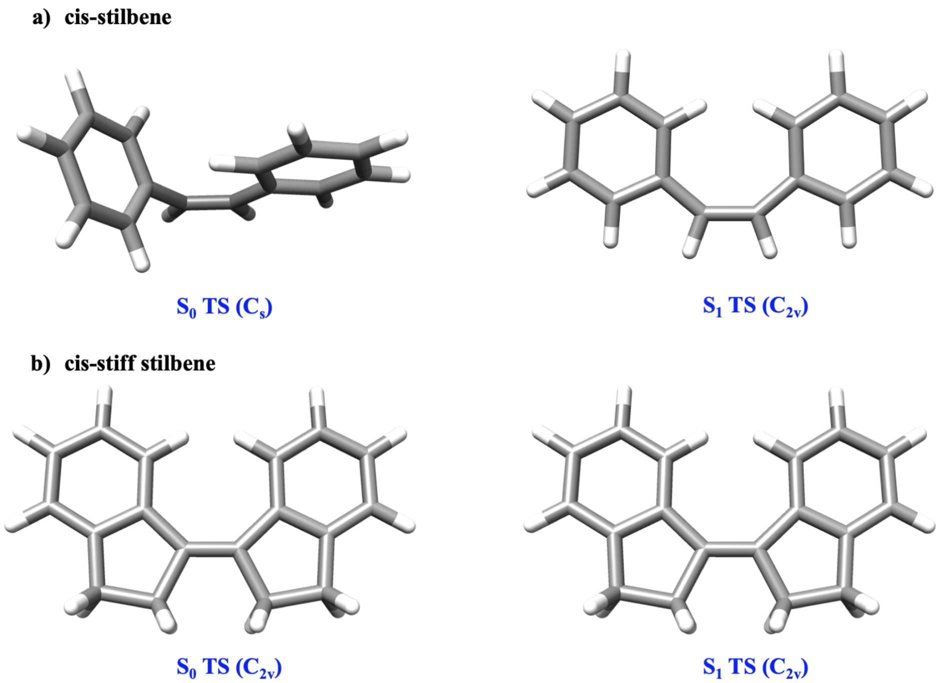

Supplementary Figure 1. Transition state structures of the *P-M* helical inversion process for a) cis-stilbene and b) cis-stiff-stilbene on the ground (S_0) and first excited (S_1) electronic state computed at B3LYP-D3/def2-TZVP(-f), and TD-B3LYP-D3/def2-TZVP(-f) levels of theory.

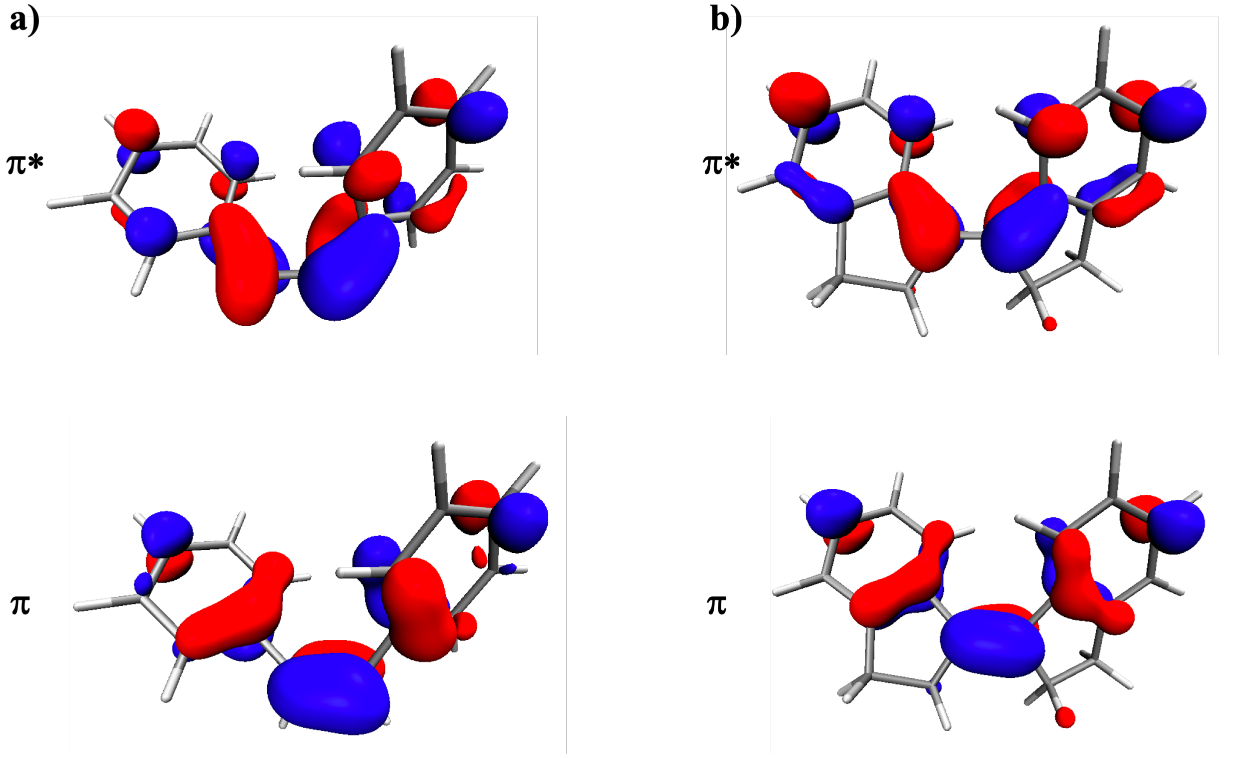

Supplementary Figure 2: HOMO (π) and LUMO (π*) of *cis*-stilbene (a) and cis-stiff-stilbene (b) at the Franck–Condon point calculated with SA-2-CAS(2,2)SCF and αFOMO-CAS(2,2)CI level of theory

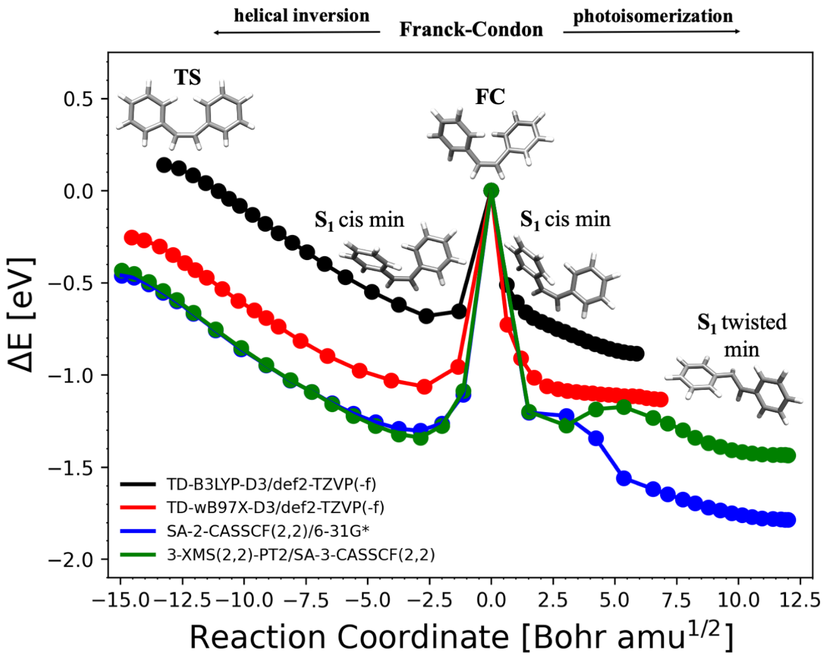

Supplementary Figure 3. Cis-stilbene potential energy surface. NEB calculations on S_1 between the Franck-Condon point and the planar transition state (left side), and between the Franck-Condon point and twisted S_1 minimum. Calculations have been performed at TD-B3LYP-D3/def2-TZVP(-f), TD-ωB97X-D3/def2-TZVP(-f), SA-2-CASSCF(2,2)/6-31G* between critical points fully relaxed at the same level of theory. The 3-XMS-(2,2)- PT2/SA-3-CASSCF(2,2)/6-31G* curve is obtained by single point calculation on the SA-2-CASSCF(2,2)/6-31G* geometries. The reaction coordinate is given in mass-weighted cartesian coordinates.

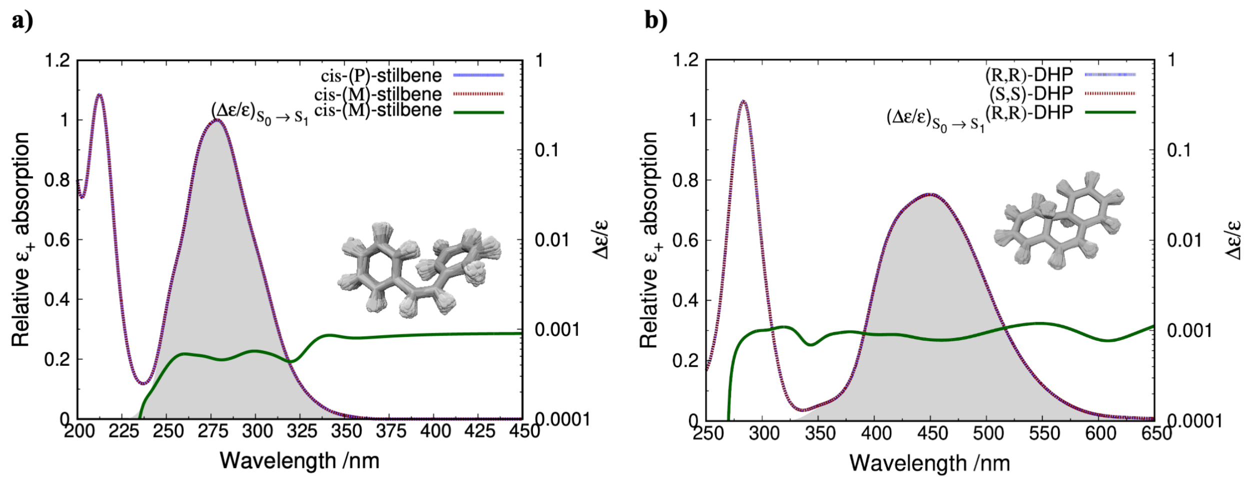

Supplementary Figure 4: a) Relative absorption of right-circularly polarized light (r-CPL) by the *(P)*- and *(M)*-enantiomers of cis-stilbene (blue and red line, respectively). The anisotropy factor (g_{stil}) for the $S_0 \rightarrow S_1$ excitation is reported in green. **b)** Relative absorption of r-CPL light by the *R,R*-DHP and *S,S*-DHP enantiomers (blue and red line, respectively). The anisotropy factor (g_{DHP}) is reported in green. The absorption spectra of the two enantiomers are graphically indistinguishable (blue and red lines overlap to give the purple line) as a result of the small value of the *g* factor.

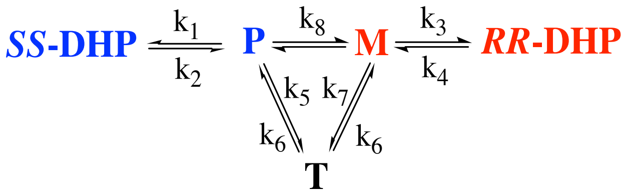

Supplementary Figure 5: Kinetic scheme used in kinetic modelling

a)

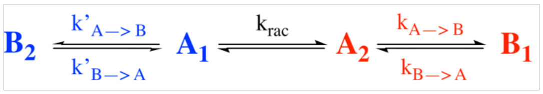

b)

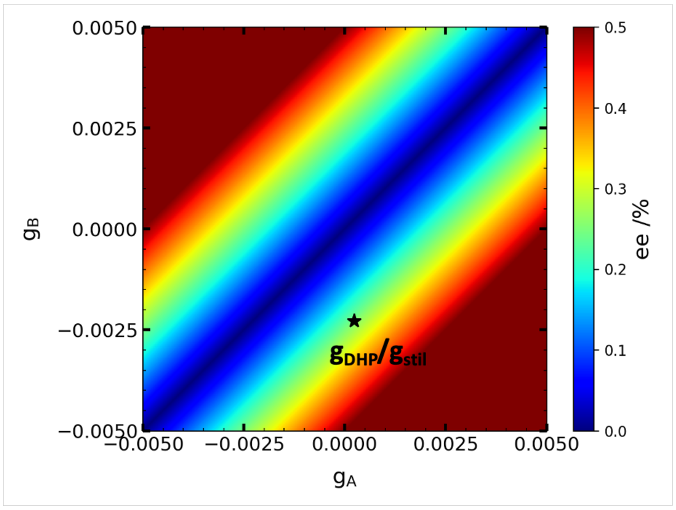

Supplementary Figure 6: a) Kinetic model employed to describe a generic photoreaction A → B where both A and B exist in two enantiomeric forms A_1/A_2 and B_1/B_2. **b)** Enantiomeric excess at the photo-stationary state for a generic photoreaction A → B as function of the anisotropy factors g_A and g_B (the *ee* can be simulated considering $k_{cis \rightarrow trans} = 0$ in eq. 1). The *g* factors of stilbene and DHP at 250 nm are also reported. The optimal condition to maximize the *ee* is reached when one enantiomer is preferentially formed and the other destroyed by the same monochromatic CPL irradiation (corresponding to *g* factors with opposite sign).

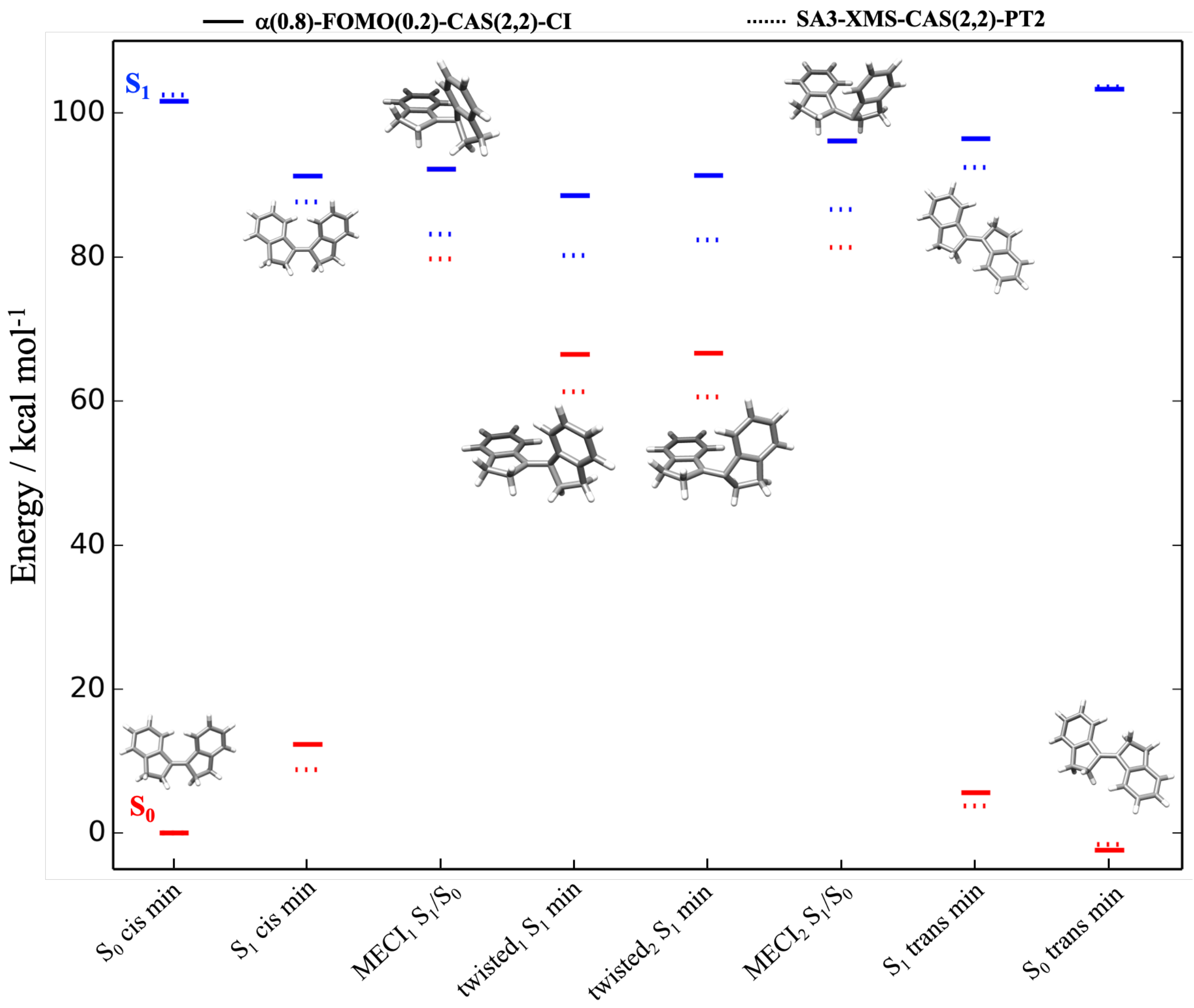

Supplementary Figure 7. Critical points along the cis-trans photoisomerization coordinate for stiff-stilbene computed at the α(0.8)-FOMO(β=0.2)-CAS(2,2)CI/6-31G* level of theory and compared to single-point energy calculations at Extended Multistate Perturbation Theory to 2nd order (XMSPT2) with two electrons in two orbitals for the three lowest energy singlet states (SA3-XMS-CAS(2,2)-PT2/6-31G*).

a) **b)**

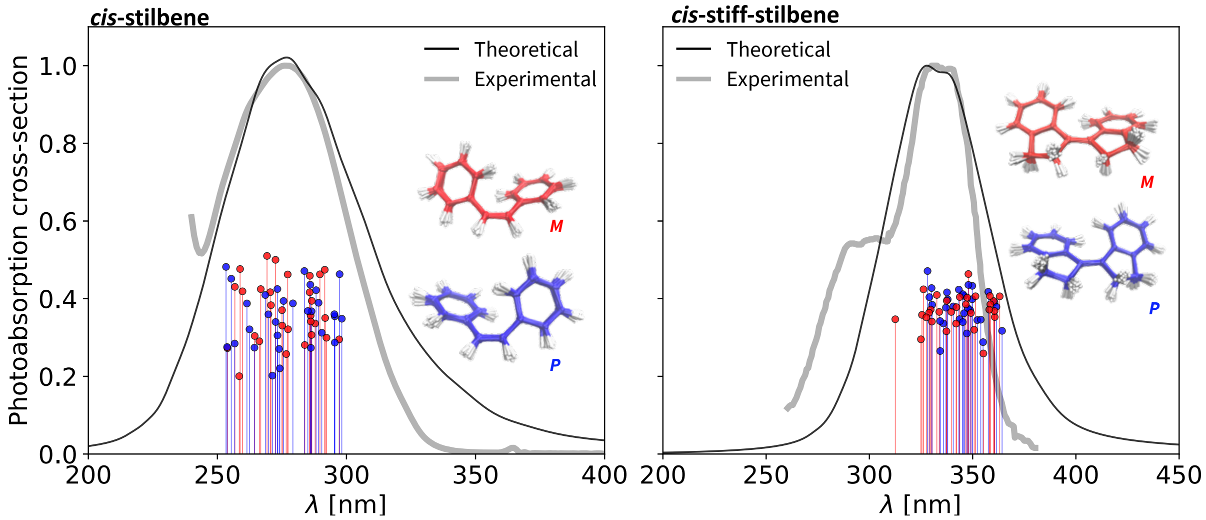

Supplementary Figure 8. The ultraviolet (UV) electronic absorption spectrum of **a)** cis-stilbene and **b)** cis-stiff-stilbene. The spectra were generated from 500 geometries sampled from a 0K harmonic Wigner distribution corresponding to the frequencies of their S_0 minima computed with B3LYP/6-31G* and B3LYP/6-31G** for cis-stilbene and cis-stiff-stilbene, respectively. The 30 initial conditions for each of the *P* and *M* enantiomers used in the AIMS trajectories are shown as blue and red lines, respectively, scaled by their oscillator strengths.

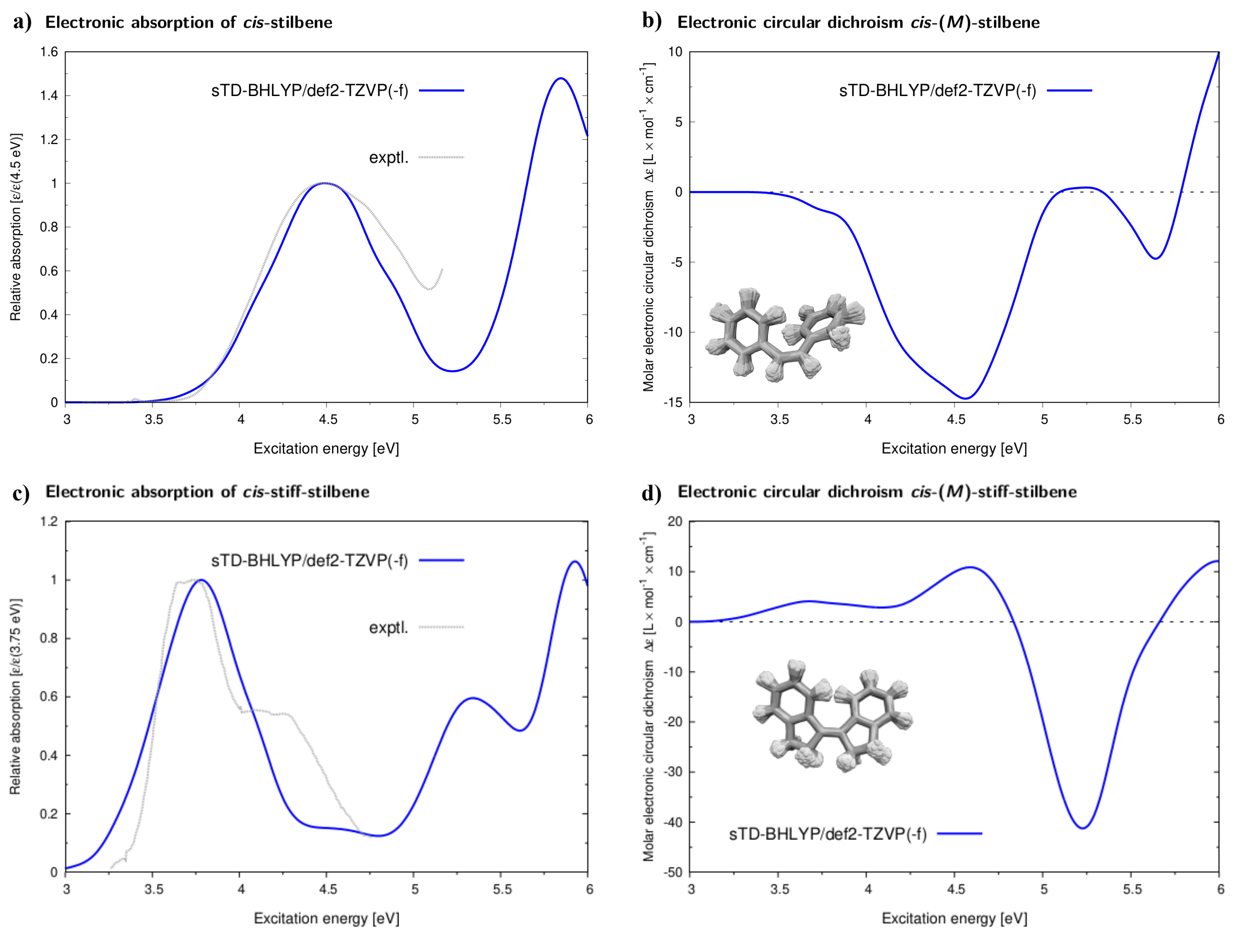

Supplementary Figure 9. Electronic absorption and circular dichroism (ECD) spectra for cis-stilbene (**a)** and **b)**) and cis-stiff-stilbene (**c)** and **d)**) computed with sTD-DFT. The spectra were generated from 1000 geometries for both molecules sampled to a 0 K harmonic Wigner distribution.

Supplementary References

1. Grimme, S. A simplified Tamm-Dancoff density functional approach for the electronic excitation spectra of very large molecules. *J. Chem. Phys.* **138**, 244104, (2013).
2. Bannwarth, C. & Grimme, S. A simplified time-dependent density functional theory approach for electronic ultraviolet and circular dichroism spectra of very large molecules. *Comp. Theo. Chem.* **1040-1041**, 45-53, (2014).
3. Becke, A. D. Density-functional exchange-energy approximation with correct asymptotic behavior. *Phys. Rev. A* **38**, 3098-3100, (1988).
4. Lee, C., Yang, W. & Parr, R. G. Development of the Colle-Salvetti correlation-energy formula into a functional of the electron density. *Phys. Rev. B* **37**, 785-789, (1988).
5. Becke, A. D. A new mixing of Hartree‐Fock and local density ‐ functional theories. *J. Chem. Phys.* **98**, 1372-1377, (1993).
6. Weigend, F. & Ahlrichs, R. Balanced basis sets of split valence, triple zeta valence and quadruple zeta valence quality for H to Rn: Design and assessment of accuracy. *Phys. Chem. Chem. Phys.* **7**, 3297-3305, (2005).
7. https://www.chemie.uni-bonn.de/pctc/mulliken-center/grimme/software/stda Accessed December 20, 2021.
8. Taniguchi, M. & Lindsey, J. S. Database of Absorption and Fluorescence Spectra of >300 Common Compounds for use in PhotochemCAD. *Photochem. Photobio.* **94**, 290-327, (2018).
9. Schellman, J. A. Circular dichroism and optical rotation. *Chem. Rev.* **75**, 323-331, (1975).
10. Weir, H., Williams, M., Parrish, R. M., Hohenstein, E. G. & Martínez, T. J. Nonadiabatic Dynamics of Photoexcited cis-Stilbene Using Ab Initio Multiple Spawning. *J. Phys. Chem. B* **124**, 5476-5487, (2020).
11. Quenneville, J. & Martínez, T. J. Ab Initio Study of Cis−Trans Photoisomerization in Stilbene and Ethylene. *J. Phys. Chem. A* **107**, 829-837, (2003).
12. Kovalenko, S. A., Dobryakov, A. L., Ioffe, I. & Ernsting, N. P. Evidence for the phantom state in photoinduced cis–trans isomerization of stilbene. *Chem. Phys. Lett.* **493**, 255-258, (2010).
13. Greene, B. I. & Farrow, R. C. Subpicosecond time resolved multiphoton ionization: Excited state dynamics of cis ‐ stilbene under collision free conditions. *J. Chem. Phys.* **78**, 3336-3338, (1983).
14. Quick, M., Berndt, F., Dobryakov, A. L., Ioffe, I. N., Granovsky, A. A., Knie, C., Mahrwald, R., Lenoir, D., Ernsting, N. P. & Kovalenko, S. A. Photoisomerization Dynamics of Stiff-Stilbene in Solution. *J. Phys. Chem. B* **118**, 1389-1402, (2014).
15. Improta, R. & Santoro, F. Excited-State Behavior of trans and cis Isomers of Stilbene and Stiff Stilbene: A TD-DFT Study. *J. Phys. Chem. A* **109**, 10058-10067, (2005).
16. Liu, F. & Morokuma, K. Computational Study on the Working Mechanism of a Stilbene Light-Driven Molecular Rotary Motor: Sloped Minimal Energy Path and Unidirectional Nonadiabatic Photoisomerization. *J. Amer. Chem. Soc.* **134**, 4864-4876, (2012).
